# Presented Loving Cup to Dr. J. F. Y. Paine

**Published:** 1910-07

**Authors:** 


### Presented Loving Cup to Dr. J. F. Y. Paine.

Upon the occasion of his last appearance before the senior class in medicine at the State Medical College, Dr. J. F. Y. Paine, Professor of Obstetrics and Gynecology at the Medical College since its establishment, was tendered a touching testimonial by the thirty-five members of the class. Dr. Paine has occupied the warmest spot in the hearts of his students, and in his departure from the Faculty the college has lost one of its most able members. The class presented him with a handsome silver loving cup, upon which was engraved the names of the thirty-five members. The cup, about twelve inches in height, mounted on an ebony base, bears the following inscription: "Presented to Dr. John F. Young Paine, Professor of Obstetrics and Gynecology, University of Texas," and on the other side: "Presented by senior class," following which are the names of the class members.

The presentation speech was made by A. O. Singleton at the end of the last lecture. In presenting the loving cup, he said:

"Doctor Paine, since our class work is drawing to an end, and as this is probably your last active work as a teacher in this school, members of the senior class were not willing that the opportunity should pass without some word of expression of their appreciation of your patience with us and your untiring efforts in our behalf. We fully realize that this has been a trying year for you, and for this reason are the more appreciative.

"Fellow students, the vacancy in the chair of obstetrics, with the resignation of its present incumbent, is one of the most serious losses that the University of Texas has ever suffered. Dr. Paine was here at the beginning of the Medical Department and no other man has done more for its upbuilding. Beginning, as it did, with practically nothing, and receiving but little aid from the outside, its present condition could not have been possible without the help of such men. And the present position which the University commands in the university world would not be possible without such a medical department. Throughout the State of Texas, throughout the United States, among the medical centers of the West and the East, the name of Dr. J. F. Y. Paine calls to the minds of the men of the profession the Medical Department of the University at Galveston.

"As the end approaches, the happiness which we should mutually feel, knowing that the drudgery of the student is ending, is overshadowed by a cloud of sadness when we realize that this is our last meeting together and also our last meeting in class work with Dr. Paine. And he, no doubt, feels this more than we. Why should he feel sad? He has given the most active part of his life to his State and people, to their greatest institution, and he has measured up to the highest standard which is required of man, which is success in his life work. What more should he desire in order to be content, and we should want for no more than such an example to keep before us as we go into the struggle.

"Doctor Paine, we desire that you accept from us a small remembrance, this loving cup, and let it remind you in the years to come that each of us holds in his heart for you the highest appreciation, the greatest admiration and the sincerest affection. May your remaining years be many and may each year be filled only with sunshine and happiness."

Dr. Paine christened the loving cup the following day. He spoke to the class at length, and was visibly affected at the parting. The cup was filled to the brim and passed among the class members. Dr. Paine reviewed the past year with the seniors and his connection with the State Medical College since its establishment at Galveston. His words were those of cheer and comfort, although sad at the parting.

The names of the class members engraved upon the cup were as follows:

J. R. Frobese, R. A. Farmer, H. W. Ogilvie, B. O. Thrasher, T. K. Jamison, C. S. Gates, Jr., Caleb O. Terrell, C. F. Smith, Elam Scull, T. S. Edwards, C. M.. Hock, Ed. Shipman, F. Gilbert, Thad Shaw, Q. B. Lee, Thomas E. Mangum, Talbert M. Hall, George W. Edgerton, Jr., T. E. Payne, Thomas W. Grice, Kenneth M. Lynch, Harry L. Leap, Edward F. Mikeska, E. W. Cavenes, Mary C. Harper, W. C. Fisher, Jr., Stephen F. Kubala, S. N. Key, A. O. Singleton, John H. Thorne, W. F. Hasskarl, G. D. Bryson, W. H. Warren, Louis Daily, C. C. Bradford.—*Galveston News.*

---

Not infrequently shifting dullness in the flanks is the only differential signs between diaphragmatic pleurisy (and beginning pneumonia) and appendicitis.—*American Journal of Surgery.*

